# Incidence and outcome of convulsive status epilepticus in Kenyan children: a cohort study

**DOI:** 10.1016/S1474-4422(07)70331-9

**Published:** 2008-02

**Authors:** Manish Sadarangani, Claire Seaton, J Anthony G Scott, Bernhards Ogutu, Tansy Edwards, Agnes Prins, Hellen Gatakaa, Richard Idro, James A Berkley, Norbert Peshu, Brian G Neville, Charles R Newton

**Affiliations:** a Centre for Geographic Medicine Research—Coast (CGMRC), Kenya Medical Research Institute (KEMRI), Kilifi, Kenya; b Nuffield Department of Clinical Medicine, University of Oxford, UK; c Department of Epidemiology and Public Health, London School of Hygiene and Tropical Medicine, London, UK; d Academic Medical Centre, University of Amsterdam, Netherlands; e Institute of Child Health, University College London, UK

## Abstract

**Background:**

Convulsive status epilepticus (CSE) is the most common neurological emergency in childhood and is often associated with fever. In sub-Saharan Africa, the high incidence of febrile illnesses might influence the incidence and outcome of CSE. We aimed to provide data on the incidence, causes, and outcomes of childhood CSE in this region.

**Methods:**

Between March, 2006, and June, 2006, we studied all children who had been admitted with CSE to a Kenyan rural district hospital in 2002 and 2003. Confirmed CSE had been observed directly; probable CSE was inferred from convulsions on arrival, requirement for phenobarbital or phenytoin, or coma with a recent history of seizures. We estimated the incidence with linked demographic surveillance, and risk factors for death and neurological sequelae were analysed by multivariable analysis.

**Findings:**

Of 388 episodes of CSE, 155 (40%) were confirmed CSE and 274 (71%) were caused by an infection. The incidence of confirmed CSE was 35 (95% CI 27–46) per 100 000 children per year overall, and was 52 (21–107) and 85 (62–114) per 100 000 per year in children aged 1–11 months and 12–59 months, respectively. The incidence of all CSE was 268 (188–371) and 227 (189–272) per 100 000 per year in these age-groups. 59 (15%) children died in hospital, 81 (21%) died during long-term follow-up, and 46 (12%) developed neurological sequelae. Mortality of children with confirmed CSE while in hospital was associated with bacterial meningitis (adjusted relative risk [RR]=2·6; 95% CI 1·4–4·9) and focal onset seizures (adjusted RR=2·4; 1·1–5·4), whereas neurological sequelae were associated with hypoglycaemia (adjusted RR=3·5; 1·8–7·1) and age less than 12 months (adjusted RR=2·5; 1·2–5·1).

**Interpretation:**

Prevention of infections and appropriate early management of seizures might reduce the incidence and improve the outcome of CSE in children in sub-Saharan Africa.

## Introduction

Convulsive status epilepticus (CSE) is the most common childhood neurological emergency in developed countries and can lead to neurocognitive sequelae and death.^1^, ^2^ The incidence of CSE in London, UK, has been reported as 18–20 per 100 000 per year in children of less than 16 years old, with higher rates in the first few years of life.^3^ These rates were similar in other studies in developed countries.^4^

In the London study, one third of childhood CSE was attributable to febrile seizures with a benign short-term outcome.^3^ However, 19% (18/95) of children with CSE associated with fever had a CNS infection; acute bacterial meningitis was particularly common and was associated with a higher risk of mortality.^3^ Seizures with fever are common in children admitted to hospitals in sub-Saharan Africa,^5^ particularly in malaria-endemic areas where CSE is well recognised. Furthermore, socioeconomic deprivation and non-white ethnic origin were independently associated with an increased incidence of CSE in the London study (Chin R, personal communication). These factors suggest a higher incidence would be expected in sub-Saharan Africa than in London. Finally, restricted access to antiepileptic drugs, particularly parenteral phenobarbital,^6^ might lead to higher mortality and morbidity in this region.

We assessed the incidence of CSE in children admitted to a rural Kenyan hospital in a malaria-endemic area. We also examined the clinical and laboratory features of these children, and identified risk factors for both death and neurological sequelae.

## Methods

### Patients and procedures

Kilifi District Hospital (KDH) is located on the Kenyan coast and has two seasonal peaks for malaria admissions, in January and July. KDH is the main district-level government inpatient facility, serving a community of about 100 000 children. Staff in a Kenya Medical Research Institute (KEMRI) Centre at the hospital provide clinical cover 24 h a day. For every child admitted, standard clinical and laboratory data are entered into a computer database. The hospital is situated within an 891 km^2^ demographic surveillance system (DSS) area,^7^ from which about 80% of all admissions are derived. This area was mapped in the year 2000 by fieldworkers on motorcycles and on foot: every building was registered by its global positioning system coordinates, and a census defined the resident population. All subsequent births, deaths, and migration events are monitored by fieldworkers who visit every participating household about once every 4 months. The housing register is also updated every 4 months by remapping.

We used the database of admissions to identify all children who were admitted between Jan 1, 2002, and Dec 31, 2003, who were between 1 month and 13 years of age and who had a history of seizures or a diagnosis of epilepsy, febrile seizures, or encephalopathy. We reviewed case notes of these children, and episodes of CSE were identified and classified as confirmed CSE or probable CSE. Confirmed CSE was defined according to the International League Against Epilepsy criteria^8^ (ie, a seizure that lasted for 30 min or longer, or intermittent seizures that lasted for less than 30 min from which consciousness was not regained for at least 1 h, as documented by medical or nursing staff). Probable CSE was defined by any one of the following criteria: convulsions on arrival to hospital; use of phenytoin or phenobarbital to stop seizures after the failure of two doses of the first-line medication (diazepam, paraldehyde, or both); coma, defined as a Blantyre coma score of 2 or less,^9^ on admission and a history of more than one seizure in the 30 min before presentation; or coma on admission and a history of more than ten seizures in the 24 h before presentation.

Clinicians entered medical history and examination findings at admission into the computer database using a standard form. Full blood count, malaria slide, plasma glucose, venous blood gas, and blood culture tests were done for all children at admission. A Coulter MDII-18 counter (Beckman-Coulter, Fullerton CA, USA) was used for full blood counts. For diagnosis of malaria, thick and thin blood smears were examined at ×1000 magnification. Glucose was assayed with a GM7 analyser (Analox Ltd, London, UK), and blood gas was assayed with an IL 1620 analyser (Instrumentation Laboratory, Lexington, MA, USA). Blood cultures were processed with a BACTEC 9050 system (Becton Dickinson, Franklin Lakes, NJ, USA) and examined by routine microbiological methods. All coagulase-negative staphylococci, bacilli, and micrococci were classified as contaminants. The CSF leucocyte count was recorded with a modified Neubauer counting chamber. For culture, CSF was inoculated on to plates of 7% horse-blood agar and 5% chocolate-and-blood agar and processed by standard microbiological techniques. The normal ranges used for CSF variables have been described previously.^5^

Lumbar puncture was done in children with indications of possible meningitis.^5^ Diagnoses were recorded for all patients according to the International Classification of Diseases, ninth revision.^10^ A primary clinical diagnosis of malaria was assigned if the child had *Plasmodium falciparum* asexual parasitaemia and no other causes could be identified. A diagnosis of febrile seizures was made for patients who had neither parasitaemia nor evidence of meningitis or encephalitis.

KEMRI-trained nursing staff used a standard form to document all seizures, which were most commonly recognised as motor convulsions. A standard protocol for emergency seizure management was used: children with seizures lasting more than 5 min received intravenous diazepam followed by intramuscular paraldehyde 10 min later, if required. At 20–30 min, intravenous phenobarbital was given, with intravenous phenytoin used subsequently for refractory episodes. Management of children in continuing CSE was decided on an individual basis by a senior clinician. Children with three or more seizures of less than 5 min within 1 h were given phenobarbital to prevent further seizures, because artificial ventilation was not available. All children with CSE received quinine and broad-spectrum antibiotics.

Children who had an episode of CSE in 2002 or 2003 were visited at home between March, 2006, and June, 2006, and screened with the Ten Questions questionnaire^11^, ^12^ for neurocognitive impairment. Children who were positive on this screen were invited for further assessment at KDH. If the child had died, the cause of death was established through a verbal autopsy^13^ with the parents or guardians.

### Statistical analysis

Data were recorded with EpiData (version 3.02) and analysed with Stata (version 8.0). If a child had multiple admissions to KDH with CSE during the study period, only the first episode was included. We used Pearson's χ^2^ test (two-tailed) to compare categorical variables, the Student's *t* test to compare the means of normally distributed data, and the Mann-Whitney test to compare non-parametric data.

The incidence of hospital admission with CSE was estimated for the subgroup of children who lived in the DSS area and presented at KDH between April 16, 2002, and Dec 31, 2003, because during this time these admissions were electronically linked to a DSS database. Estimates of the ages and of the numbers of children of each sex were made at the midpoint of this interval (Feb 22, 2003) by fitting a linear regression line through the population counts (on a log scale) from each of ten enumeration rounds between September, 2000, and May, 2006. Confirmed and probable cases were combined to estimate overall incidence rates. Confidence intervals and rate comparisons were calculated with Poisson regression analysis.

Risk ratios for mortality and neurological sequelae in children with confirmed CSE were calculated by binomial regression with a log link. Exploratory investigations of confounding and interacting factors were assessed with Mantel-Haenszel methods. Risk factors were entered into a multivariable model with a forward stepwise approach on the basis of the strength of the univariate associations. A likelihood ratio test was used to compare models. All variables with p<0·25 in an unadjusted univariate analysis and no interaction terms were included in the multivariable model; only those with p<0·05 were retained in the model. We decided a priori to include acute bacterial meningitis in each model because it is a direct CNS infection. We estimated unadjusted effects for all available data, and multivariable analyses were done for children for whom complete data had been collected. Children who did not survive to discharge were excluded from the analysis of sequelae.

This study was approved by the National Ethics Committee of Kenya, and parents gave consent for study staff to interview them and examine their children as part of the follow-up study.

### Role of the funding source

The sponsor of the study had no role in study design, data collection, data analysis, data interpretation, or writing of the report. The corresponding author had full access to all the data in the study and had final responsibility for the decision to submit for publication.

## Results

The figure shows the study design and profile. In the 2-year study period, 2837 (27%) of all children admitted to KDH were admitted with seizures. We obtained hospital records for 2585 (91%) children, 388 of whom had at least one episode of CSE. Of these children, 155 (40%) had confirmed CSE, and 233 (60%) had probable CSE. 17 recorded episodes of CSE in children who had multiple episodes were excluded from analysis.

**Figure:**
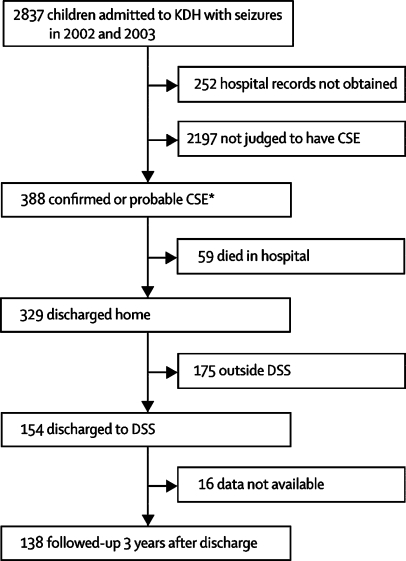
Study design and profile *All 388 CSE cases were used for descriptive analysis. The 178 cases linked to the DSS database were used for incidence analysis, and the 155 confirmed CSE cases were used for risk factor analysis.

Table 1 shows the demographic and clinical features of all children admitted with CSE, and criteria used to diagnose probable CSE. 274 (71%) cases had an infective cause. 253 (65%) children had a positive malaria slide, and malaria was the primary diagnosis for 206 (53%) children. 41 (11%) children had bacteraemia and 33 (9%) had acute bacterial meningitis. Seizures had a focal onset in 151 (39%) children, and hypoglycaemia (blood glucose <2·5 mmol/L) was detected in 41 (11%) children.

**Table 1:** Characteristics of patients in descriptive analysis

	**Confirmed CSE (n=155)**	**Probable CSE (n=233)**
**Inclusion criteria for probable CSE**		
Convulsions on arrival to hospital	..	180 (77%)
Phenytoin or phenobarbital to stop seizures	..	48 (21%)
Coma on admission, >1 seizure in previous 30 min	..	1 (<1%)
Coma on admission, >10 seizures in previous 24 h	..	4 (2%)
**Demographic characteristics**		
Age <12 months	29 (19%)	48 (21%)
Male	76 (49%)	113 (48%)
**Clinical history**		
History of any seizures	36 (23%)	83 (36%)
Presented during first seizure of illness	23 (15%)	68 (29%)
Focal onset seizure	77 (50%)	74 (32%)*
Received >2 antiepileptic drugs during CSE	63 (41%)	5 (2%)
**Physical findings during CSE**		
Malnutrition (WHZ <−2)	50 (32%)†	70 (30%)‡
Fever (axillary temperature ≥38·0°C)	74 (48%)	105 (45%)§
Hypoxia (oxygen saturation <92%)	108 (70%)	94 (40%)*
**Laboratory parameters**		
Hypoglycaemia (blood glucose <2·5 mmol/L)	18 (12%)	23 (10)¶
Severe anaemia (haemoglobin <50 g/L)	14 (9%)	21 (9%)‖
Positive malaria slide	101 (65%)	152 (65%)
Bacteraemia	23 (15%)	18 (8%)**
Lumbar puncture done	136 (88%)	179 (77%)
Acute bacterial meningitis	20 (13%)††	13 (6%)‡‡
**Primary diagnosis**		
Malaria without febrile convulsion	49 (32%)	65 (28%)
Febrile convulsion secondary to malaria	35 (23%)	57 (24%)
Febrile convulsion secondary to other infection	3 (2%)	11 (5%)
Acute bacterial meningitis	15 (10%)	16 (7%)
Other infection without febrile convulsion	10 (6%)	13 (6%)
Encephalopathy of unknown cause	24 (15%)	11 (5%)
Anaemia	11 (7%)	21 (9%)
Epilepsy	2 (1%)	21 (9%)
**Outcomes**		
Died before discharge	36 (23%)	23 (10%)
Neurological sequelae at discharge	28 (18%)	18 (8%)

WHZ=weight-for-height *Z* score.

* 4 missing values.

† 7 missing values.

‡ 18 missing values.

§ 1 missing value.

¶ 5 missing values.

‖ 2 missing values.

** 1 missing value.

†† 19 missing values.

‡‡ 54 missing values.

59 (15%) children died in hospital, 28 (47%) of these within 24 h of admission and 44 (75%) within 48 h; 81 (21%) died during the following 3 years. 46 (12%) children had neurological sequelae at discharge. Motor deficits were the most common disorder, affecting 40 (87%) children who had sequelae. Death before discharge was more common in children with confirmed CSE than in those with probable CSE (table 1; difference between groups 13%, 95% CI 6–21; p=0·0003). The proportion of children who had neurological sequelae at discharge was also higher in the group with confirmed CSE than in the group with probable CSE (table 1; difference between groups 10%, 3–17; p=0·0020).

337 children were admitted with their first episode of CSE between April 16, 2002, and Dec 31, 2003, and 178 (53%) of these resided within the DSS area. Of this group, 58 (33%) children had confirmed CSE and 120 (67%) had probable CSE. Table 2 shows the characteristics of this group of children. The mortality or development of neurological sequelae on discharge did not differ significantly between children who did or did not reside within the DSS area. Table 3 shows the incidence of admission to KDH with either confirmed or probable CSE, both overall and broken down by age-group. The incidence of CSE did not vary significantly by sex in any age-group.

**Table 2:** Characteristics of patients in incidence analysis

	**Confirmed CSE (n=58)**	**Probable CSE (n=120)**
**Inclusion criteria for probable CSE**		
Convulsions on arrival to hospital	..	102 (85%)
Phenytoin or phenobarbital to stop seizures	..	17 (14%)
Coma on admission, >1 seizure in previous 30 min	..	0 (0%)
Coma on admission, >10 seizures in previous 24 h	..	1 (1%)
**Demographic characteristics**		
Age <12 months	7 (12%)	28 (23%)
Male	28 (48%)	55 (46%)
**Clinical history**		
History of any seizures	16 (28%)	35 (29%)
Presented during first seizure of illness	12 (21%)	36 (30%)
Focal onset seizure	33 (57%)	35 (29%)*
Received >2 antiepileptic drugs during CSE	25 (43%)	4 (3%)
**Physical findings during CSE**		
Malnutrition (WHZ <-2)	18 (31%)†	32 (27%)‡
Fever (axillary temperature ≥38·0°C)	24 (41%)	53 (44%)
Hypoxia (oxygen saturation <92%)	38 (66%)	58 (48%)†
**Laboratory parameters**		
Hypoglycaemia (blood glucose <2·5 mmol/L)	5 (9%)	12 (10%)†
Severe anaemia (haemoglobin <50 g/L)	3 (5%)	11 (9%)*
Positive malaria slide	40 (69%)	86 (72%)
Bacteraemia	9 (16%)	5 (4%)†
Lumbar puncture done	52 (90%)	95 (79%)
Acute bacterial meningitis	5 (9%)§	3 (3%)¶
**Primary diagnosis**		
Malaria without febrile convulsion	21 (36%)	36 (30%)
Febrile convulsion secondary to malaria	13 (22%)	34 (28%)
Febrile convulsion secondary to other infection	2 (3%)	7 (6%)
Acute bacterial meningitis	2 (3%)	4 (3%)
Other infection without febrile convulsion	4 (7%)	2 (2%)
Encephalopathy of unknown cause	8 (14%)	7 (6%)
Anaemia	5 (9%)	13 (11%)
Epilepsy	1 (2%)	5 (4%)
**Outcomes**		
Died before discharge	11 (19%)	13 (11%)
Neurological sequelae at discharge	13 (22%)	8 (7%)

* 2 missing values.

† 1 missing value.

‡ 7 missing values.

§ 6 missing values.

¶ 25 missing values.

**Table 3:** Admission to Kilifi District Hospital because of CSE

	**In census (n)**	**Confirmed CSE**		All CSE*	
		Admitted (n)	Incidence (95% CI)†	Admitted (n)	Incidence (95% CI)†
1–11 months	7871	7	52 (21–107)	36	268 (188–371)
12–59 months	30 886	45	85 (62–114)	120	227 (189–272)
60–155 months	57 581	6	6 (2–13)	22	22 (14–34)
All ages	96 338	58	35 (27–46)	178	108 (93–125)

* Confirmed CSE plus probable CSE.

† Incidence per 100 000 child-years.

The univariate analysis showed that five factors were associated with a high risk of mortality in hospital: acute bacterial meningitis, hypoglycaemia, age of less than 12 months, bacteraemia, and focal onset seizures (table 4). A positive malaria slide was associated with a low risk of mortality in hospital. Factors not associated with mortality were sex, history of seizures, malnutrition (weight-for-height *Z* score <−2), fever, hypoxia, use of more than two antiepileptic drugs, and severe anaemia (haemoglobin <50 g/L). Acute bacterial meningitis and focal onset seizures were the only significant risk factors for death in the multivariable analysis. There was no evidence of an interaction between meningitis and focal onset of seizures (χ^2^ test of homogeneity p=0·9259).

**Table 4:** Significant risk factors for mortality and neurological sequelae following confirmed CSE

	**Univariate RR (95% CI)**	**p**	**Adjusted RR (95% CI)**	**p**
**Mortality**				
Acute bacterial meningitis*	3·071 (1·60–5·90)	0·0008	2·590 (1·36–4·93)	0·0037
Hypoglycaemia (blood glucose <2·5 mmol/L)	2·537 (1·43–4·50)	0·0014	1·930 (0·97–3·82)	0·5940
Age <12 months	2·146 (1·42–4·25)	0·0013	0·535 (0·14–1·98)	0·3482
Bacteraemia	2·207 (1·24–3·94)	0·0074	1·378 (0·53–3·61)	0·5147
Focal-onset seizure	2·302 (1·22–4·35)	0·0101	2·432 (1·09–5·41)	0·0294
Positive malaria slide	0·382 (0·22–0·68)	0·0010	0·947 (0·37–2·46)	0·9118
**Neurological sequelae**				
Hypoglycaemia (blood glucose <2·5 mmol/L)	3·333 (1·84–6·04)	0·0001	3·525 (1·75–7·10)	0·0004
Age <12 months	2·145 (1·09–4·21)	0·0264	2·473 (1·21–5·05)	0·0130
Focal onset seizure	1·991 (1·02–3·88)	0·0428	1·592 (1·37–2·46)	0·9118

* 19 missing values.

Univariate analysis identified three risk factors that were associated with an increased of sequelae: hypoglycaemia, age less than 12 months, and focal onset seizures (table 4). In the multivariable analysis, only hypoglycaemia and age less than 12 months were significant risk factors for the development of neurological sequelae.

Data were available for 138 of the 154 (90%) children who were discharged from hospital who were residing within the DSS area during their admission. 22 (16%) died after discharge—18 (82%) of these with seizures as a part of their final illness. 15 (11%) children in the DSS database had neurological sequelae, with motor and speech impairments each affecting 11 (7%) children. 16 (12%) children from this cohort had active epilepsy (at least one unprovoked seizure within 1 year of admission).

## Discussion

We have shown the incidence of admission to hospital with CSE in the Kilifi district of rural Kenya is more than that in London.^3^ If probable cases are also included, the incidence is almost eight times higher than in London. Even this higher rate, which is more likely to represent the true disease burden than is the rate of confirmed cases alone, might be an underestimate owing to incomplete ascertainment, for several reasons. First, many children with CSE in this region die without reaching hospital, and a few might be treated successfully in peripheral clinics. Second, only 178 children could be linked to the DSS database, perhaps because clerical staff were unable to obtain accurate residence information during the emergency admission—thus, we were able to include in our incidence analysis fewer children than have other recent studies in this region. Finally, we were able to obtain the case notes for only 91% of children suspected of having CSE.

Previous studies of CSE in children, most of which were done in developed countries, have reported lower incidence rates (from 3·8 to 38 per 100 000 per year)^4^ than we recorded in Kenya. Several factors are likely to explain this difference. The local population has restricted access to medical facilities and transport, and treatment before admission to hospital is often inadequate. Seizures therefore last longer than in developed countries before medical care is reached, and are thus more refractory to treatment.^14^ This factor might be more important in other regions of Africa, where local epilepsy services are not provided as they are in Kilifi. Studies in developed countries report an infectious cause in 51–66% of CSE cases,^15^, ^16^, ^17^ which is lower than our proportion of 71%. In light of this link between CSE and infection, these higher incidence rates might also reflect the high prevalence of infection in sub-Saharan Africa. Malaria is a common cause of childhood infections in this area, and could account for much of the greater incidence.

The inpatient mortality rate in this study is substantially higher than that reported in other studies of children. The short-term (within 30 days) mortality rate in a recent systematic review of population-based studies was 3–9% in children.^4^ Other studies have reported a mortality rate in children of up to 11%.^18^, ^19^ Studies in predominantly adult populations describe a mortality rate of 7–46%.^20^, ^21^, ^22^ Mortality was associated with bacterial meningitis, which has a worse outcome in this area than in developed countries^23^ and has also been associated with a poor outcome in children with CSE.^3^ Furthermore, most children who died did so within 48 h of admission; this suggests that interventions to reduce mortality should be aimed at the prevention of the major causes of CSE and at emergency seizure treatment before hospital admission.

A positive malaria slide was associated with a lower risk of mortality in the univariate analysis, possibly because children who did not have malaria had CSE with a more severe underlying cause, such as encephalitis, which was associated with a worse outcome. After adjustment for other factors in the multivariable analysis, the presence of malaria did not affect outcome significantly.

The proportion of children who had neurological sequelae at discharge is lower than in previous studies, where neurological sequelae have been reported in 28–53% of children after CSE,^17^, ^18^, ^24^, ^25^, ^26^ and the risk of epilepsy after CSE has been estimated at between 19% and 82%.^24^, ^25^, ^27^ In this study, children were assessed at discharge and no psychomotor testing was done. In a previous study in this area of Kenya, 24% of children who had complicated seizures associated with malaria in this population had neurocognitive impairment 3–9 years later.^28^ The lower rate of sequelae reported here might be due to a shorter period of follow-up and a higher mortality rate than in some previous studies.

Young age, longer duration of seizure, and acute symptomatic causes (ie, an acute underlying illness) have previously been associated with mortality and morbidity in CSE.^4^, ^15^, ^16^, ^17^, ^18^, ^25^, ^27^, ^29^, ^30^, ^31^, ^32^, ^33^ In this study, focal onset seizures were a risk factor for mortality, which suggests an underlying preventable cause.^34^ Age less than 12 months and hypoglycaemia were associated with the development of neurological sequelae, so early detection and rapid correction of hypoglycaemia might prevent some of these sequelae.

The differences between the confirmed CSE and probable CSE groups in this study might have been caused by time to admission to hospital and other immeasurable factors. Analysis of risk factors was therefore restricted to children who had confirmed CSE, to identify the most important predictors of outcome. Our classification was designed to capture children who would otherwise have been missed owing to delayed presentation to hospital. In a resource-poor setting, this delay means that many children would be expected to have a prolonged seizure before their arrival at hospital. This suggestion is supported by a recent study of prolonged seizures in Malawi, in which the average duration of a seizure before hospital treatment was 2 h.^35^ Furthermore, in an epidemiological study of epilepsy in the same population of children in Kenya, many children had a history of CSE lasting many hours.^36^ This supports the inclusion of probable cases to assess the burden of CSE, but suggests that children in the confirmed group have more severe illness.

The incidence of childhood CSE in this region of Kenya is higher than reported elsewhere and has a worse outcome, resulting in a substantial public health issue. Infections such as malaria, inadequate treatment before arrival at hospital and difficulties in reaching hospital might have contributed to this high incidence and mortality. Cheap and effective antiepileptic drugs such as phenobarbital are available, but the withdrawal of parenteral phenobarbital by pharmaceutical companies^6^ might aggravate the poor outcome of CSE in sub-Saharan Africa. Community education, prevention of malaria and bacterial meningitis, and treatment of seizures and correction of hypoglycaemia in local clinics might improve the outcome of children with CSE in future.
